# A novel *ZNF699* mutation in a patient with DEGCAGS syndrome and severe B cell depletion

**DOI:** 10.70962/jhi.20250204

**Published:** 2026-07-30

**Authors:** Giuliana Giardino, Roberta Romano, Gabriella Maria Squeo, Elisabetta Toriello, Antonio De Rosa, Antonio Ammendola, Giorgia Gemma, Jessica Rzasa, Jen Kerkhof, Eleonora di Venere, Mariateresa Cantelli, Emilia Cirillo, Vincenzo Nigro, Bekim Sadikovic, Claudio Pignata, Giuseppe Merla

**Affiliations:** 1 Department of Translational Medical Sciences, Pediatric Section, Federico II University of Naples, Naples, Italy; 2 Department of Molecular Medicine and Medical Biotechnology, University of Naples Federico II, Naples, Italy; 3 Laboratory of Regulatory and Functional Genomics, Fondazione IRCCS Casa Sollievo della Sofferenza, San Giovanni Rotondo, Italy; 4 Department of Science and Technology, University of Sannio, Benevento, Italy; 5 Molecular Diagnostics Program, and Verspeeten Clinical Genome Centre, London Health Sciences Centre, London, Canada; 6 Department of Pathology and Laboratory Medicine, Western University, London, Canada; 7 Dermatology Unit, Department of Mental and Physical Health and Preventive Medicine, Università degli Studi della Campania “Luigi Vanvitelli”, Naples, Italy; 8 Medical Genetics, Department of Precision Medicine, Università degli Studi della Campania “Luigi Vanvitelli”, Naples, Italy

## Abstract

We report the first detailed immunological characterization of a DEGCAGS patient, showing that biallelic *ZNF699* loss-of-function variants can cause syndromic combined immunodeficiency and that DNA methylation profiling improves diagnostic precision in selected inborn errors of immunity.

## Graphical Abstract

**Figure:**
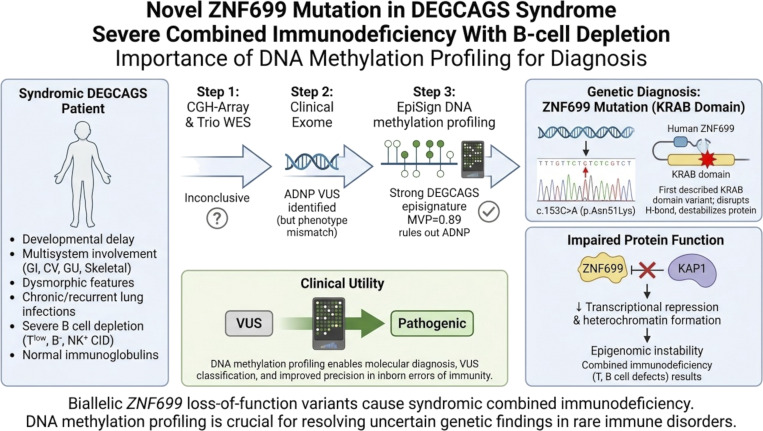

## Introduction

Developmental delay with gastrointestinal, cardiovascular, genitourinary, and skeletal abnormalities syndrome (DEGCAGS, MIM #619488) syndrome is a rare autosomal recessive disorder caused by biallelic loss-of-function *ZNF699* variants (1). Immunodeficiency and/or recurrent infections are reported in 42.3% of patients (1). A specific DNA methylation episignature has been described for this syndrome. We report a 20-year-old male with combined T^low^B^−^ immunodeficiency, characterized by recurrent severe lung infections and marked B cell depletion, in whom genetic diagnosis was supported by genome-wide DNA methylation profiling.

## Results

A 13-year-old male born to healthy consanguineous Moroccan parents came to our attention for recurrent upper and lower respiratory infections requiring frequent antibiotic treatments and hospital admissions, mild obstructive sleep apnea, and asthma. Family history was unremarkable. High-resolution computed tomography scan revealed bilateral diffuse bronchiectasis, bronchiolectasis, and obliterans bronchiolitis. Spirometry revealed a mixed obstructive and restrictive pattern, while no alterations were identified at the 6-min walking test. Clinical examination revealed premature hair graying, sunken and hypermetropic eyes, hypotelorism, small dysmorphic ears, beak-shaped nose, nail dystrophy, hyperpigmentation of the neck and periumbilical and axillary regions, obesity, stature at the lowest limit of the midparental height, hypovirilization and gynecomastia, joint hypermobility, brachydactyly of the fifth digit, second and third toe syndactyly, and splenomegaly. He also had a history of cryptorchidism, feeding difficulties, hypotonia, developmental delay and borderline IQ score (IQ 77), and atopic dermatitis with occasional fungal and bacterial superinfection. Other features included bilateral pyelectasis, left ventricular hypertrabeculation, hypermetropia, increased retinal vessel tortuosity, and hyperemic optic disc with blurred margins.

The immunological work-up revealed a significant reduction of CD4^+^CD45RA^+^ naïve T cells (12%), CD8^+^CD45RA^+^ naïve T cells (42%), and CD19^+^ B cells (2%, 33.2 cells/μl). Proliferative response to mitogens and κ-deleting recombination excision circle levels were impaired, whereas T cell receptor excision circle, T cell receptor repertoire, anti-pneumococcal response, total and specific immunoglobulins, and isohemagglutinins levels were within normal ranges. A rectal mucosa biopsy revealed CD138^+^CD20^+^CD21^+^IgG^+^/IgA^+^/IgM^+^ B cells. Based on infectious history and immunological findings, a diagnosis of combined immunodeficiency (CID) was made according to European Society for Immunodeficiencies (ESID) criteria. Detailed description of the immunological features is summarized in Table 1.

**Table 1.** Immunological features at different ages

Age	13 years	17 years	19 years	Reference value [Normal ranges]
WBC (cells/mmc)	3,970	6,290	4,380	​
Lymphocytes (cells/mmc)	2,160	2,520	1,660	1,340–3,173
CD3^+^ % (cells/mmc)	**61 (1,317)**	64 (1,613)	67	62.6–80.4
CD4^+^ % (cells/mmc)	38 (820)	35 (882)	38	32.6–51.5
CD8^+^ % (cells/mmc)	**17 (367)**	22 (554)	21	19.0–29.0
CD19^+^ % (cells/mmc)	**2 (63)**	**2 (50)**	**2 (33)**	11.9–21.0
CD56^+^ % (cells/mmc)	**28 (605)**	**32 (806)**	**31 (515)**	4.3–16.2
CD3^+^HLADR^+^ % (cells/mmc)	**15 (198)**	**11 (177)**	8	2.3–8.6
CD3^+^CD4^+^CD45RA^+^ % (cells/mmc)	**13 (107)**	**9 (79)**	**12 (76)**	40.9–65.7
CD3^+^CD4^+^CD45RO^+^ % (cells/mmc)	**87 (713)**	**91 (803)**	**88 (555)**	25.1–52.1
CD3^+^CD8^+^CD45RA^+^ % (cells/mmc)	**29 (106)**	**44 (244)**	**42 (146)**	​
CD3^+^CD8^+^CD45RO^+^ % (cells/mmc)	**61 (224)**	**56 (310)**	**58 (202)**	​
TCRγδ^+^CD3^+^ % (cells/mmc)	-	7 (113)	7 (78)	3.3–10.0
TCRαβ^+^CD3^+^CD4^−^CD8^−^ (%)	-	0.4 (6)	-	0.57–5
Naïve B cells CD19^+^IgD^+^CD27^−^ % (cells/mmc)	-	**25 (12)**	**72 (24)**	75.2–86.7
Non-switched memory B cells CD19^+^IgD^+^CD27^+^ % (cells/mmc)	-	**40 (20)**	9 (3)	4.6–10.2
Switched memory B cells CD19^+^IgD^−^CD27^+^ % (cells/mmc)	-	**27 (13)**	**19 (6)**	3.3–9.6
Transitional B cells CD19^+^IgM^++^CD38^++^ % (cells/mmc)	-	2 (1)	2.3 (0.8)	0.7–24
Class-switched plasmablasts CD19^+^IgM^−^/^+^CD38^+++^ % (cells/mmc)	-	6 (3)	**0 (0)**	0.7–6
CD21^low^ B cells CD19^+^CD21^low^CD38^−^ % (cells/mmc)	-	**7 (3.5)**	-	0.9–3.3
TRECs	Normal levels	Normal levels	Normal levels	-
KRECs	**Low levels**	**Low levels**	**Low levels**	-
TCRVb repertoire	-	Normal	-	-
Proliferative response	**PHA 15%, PKW 10%, PMA 10% **	​	​	>30% of the control
IgG/IgA/IgM (mg/dL)	1,070/287/62.5	939/317/194	1,180/397/95	604–1,909/61–301/59–297
Specific antibody response	IgG positive for CMV IgG, HSV, rubella, VZV, EBV, Parvovirus B19	-	-	-
Anti-pneumococcus antibodies	-	81 mg/L	-	>35 mg/L
HIV	Negative	-	-	Negative

Values highlighted in bold are outside the age-specific reference interval.

WBC, white blood cells; TRECs, T cell receptor excision circles; KRECS, κ-deleting excision circles; TCRVb, T cell receptor Vb; PHA, phytohemoagglutinin; PKW, pokeweed; PMA, Phorbol 12-myristate 13-acetate; CMV, cytomegalovirus; HSV, herpes simplex virus; VZV, varicella zoster virus; EBV, Epstein-Barr virus; HIV, human immunodeficiency virus.

**Figure 1.**
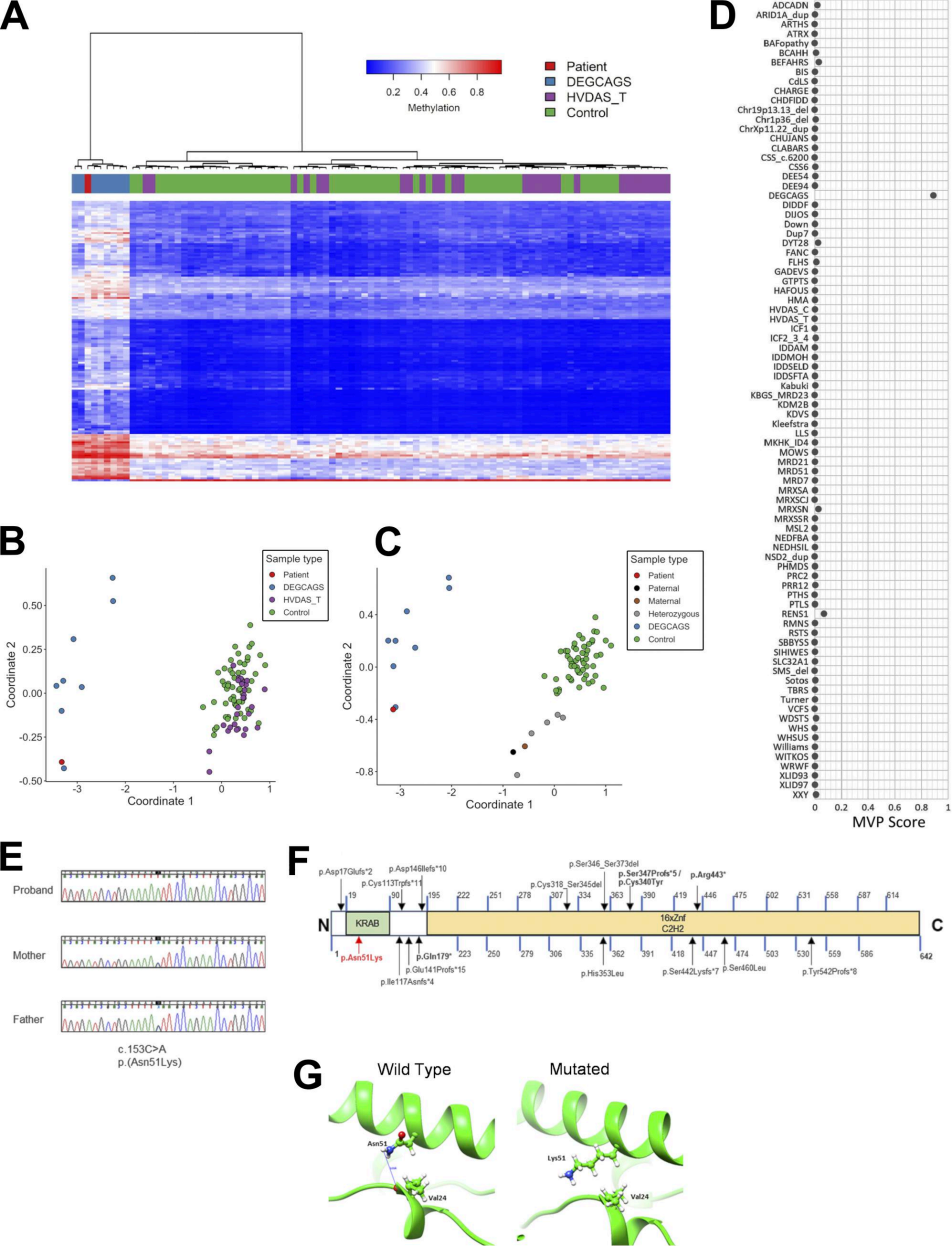
**Immunological evaluations at different time points and EpiSign (DNA methylation) analysis of peripheral blood from a case with a VUS missense ZNF699 variant, the causative gene for DEGCAGS syndrome. (A and B)** (A) Hierarchical clustering showing the methylation levels (βvalues) of the most differentially methylated probes across the genome that best discriminate the disorder from all other reference conditions, and (B) multidimensional scaling plots indicate the case (red) has a DNA methylation profile similar to subjects with a confirmed DEGCAGS episignature (blue) and distinct from HVDAS_T patients (purple) and controls (green). **(C)** Parents’ (black, father, and brown, mother) episignature clusters with heterozygous carriers (gray). **(D)** MVP score, a multi-class supervised classification system capable of discerning between multiple episignatures by generating a probability score for each episignature. The elevated score for DEGCAGS shows an episignature similar to the DEGCAGS syndrome reference. **(E)** The DNA electropherogram from Sanger sequencing reports the c.153C>A mutation in the ZNF699 gene present in our case inherited from heterozygous parents. **(F)** KRAB domain and 16 ZFs. Domain architectures of human ZNF699 protein were given by UniProt Q32M78. The numbers showed up and down of the figure indicate which amino acid are defining the start and stop of the protein, the start and stop of the KRAB domain, and the start of the 16 ZF domains. The compound heterozygous variants are highlighted in bold. The variant described in this paper is highlighted in bold red. **(G)** Visualization of the KRAB domain using the predicted structure of the mutated protein, modeled by AlphaFold2 and rendered in UCSF Chimera 1.18. The mutation leads to the disruption of a hydrogen bond between the amino acid residues at positions 51 and 24, which is intact in the wild-type protein.

Array comparative genomic hybridization did not reveal structural chromosomal alterations, and fragile-X and Cohen syndrome were excluded by Sanger sequencing. In 2018, whole exome sequencing (WES) was non-informative, and in 2022, clinical exome sequencing analysis revealed a de novo heterozygous variant (c.817C>T) in *ADNP* gene causing Helsmoortel-Van der Aa syndrome (HVDAS). The variant was classified on ClinVar as a variant of uncertain significance (VUS). Since the patient’s phenotype was only partially consistent with HVDAS and an episignature for HVDAS was already established (2), we performed EpiSign analysis to reassess the VUS. Patient’s methylation profile did not match the reported HVDAS episignature (Fig. 1, A and B), while a high confidence match with an methylation variant pathogenicity (MVP) of 0.891 was found for the episignature associated with DEGCAGS (3) (Fig. 1 D). Sanger sequencing identified a homozygous missense variant (c.153C>A; p. Asn51Lys) in the *ZNF699* gene with parents carrying the same variant in heterozygous state (Fig. 1 E). As expected from their genotype, the parents’ episignature is classified within the heterozygous carrier cluster (Fig. 1 C). This variant, classified as a VUS (PM2+PP4+BP4) by Franklin (Genoox), results in the substitution of asparagine at position 51 with lysine. This change disrupts the interaction between asparagine and valine, which is facilitated by a hydrogen bond in the wild-type structure (Fig. 1 G). The AlphaMissense score yielded a value of 0.948, indicating a high likelihood that the amino acid change is damaging to the protein’s function. The variant falls within the Krüppel-Associated Box (KRAB) domain, which plays a critical role in transcriptional repression (Fig. 1 F). The lack of the hydrogen stabilizing bond could lead to altered molecular interactions or affect the protein’s ability to interact with other biomolecules, potentially compromising its biological activity. To date, 15 different *ZNF699* variants have been identified in 30 individuals (3), mostly located in the C2H2 zinc fingers (ZFs) domain or in the region between this domain and the KRAB domain. The c.153C>A missense variant identified in our patient is the first within the KRAB domain.

## Discussion

We report on a syndromic patient in whom a diagnosis of DEGCAGS was suggested by the EpiSign analysis and confirmed by the identification of a homozygous *ZNF699* variant. The diagnosis was not previously achieved through genetic analysis since DEGCAGS syndrome was associated with *ZNF699* only in 2021 (1); thus, it was not highlighted in the first WES analysis performed in 2018, and it was not yet included in the list of 5,228 genes of the clinical exome performed in 2022. This evidence further emphasizes the importance of reanalysis of results of genetic testing over time. In the last few years, genome-wide DNA methylation analysis has been used to identify specific episignatures in about 100 genetic diseases, mainly neurodevelopmental disorders. Epigenomics is a promising strategy for resolving VUS, especially when experimental models or functional assays are unavailable, as in the cases of *ZNF699* and *ADNP*.

Interestingly, many conditions for which a specific episignature has been identified also show signs of immunodeficiency supporting the hypothesis of a potential contribution of epigenetics in the pathophysiology of certain inborn errors of immunity (IEIs) (4). Episignatures may support genetic diagnosis and VUS validation, and the identification of specific DNA methylation profile for different IEIs might significantly expedite the diagnostic process of unresolved cases also in the field of IEIs. Although whole-blood methylation profiles can be influenced by leukocyte composition, the EpiSign classifier has been validated across a wide range of hematologic and immunologic backgrounds and remains robust even in disorders characterized by marked lymphopenia (5).

The variant identified in this study is classified as a VUS. Bioinformatic analysis predicted that the aminoacidic change leads to a destabilization of the secondary structure of the protein, which may affect its ability to interact with other biomolecules. Although in silico protein modeling approaches such as AlphaFold cannot conclusively establish the pathogenicity of this VUS and no validated experimental system is currently available to functionally assess ZNF699, the presence of a gene-specific episignature provides an orthogonal and widely accepted line of evidence to support pathogenicity in disorders associated with aberrant DNA methylation (3).

Our patient shows very peculiar and persistent immunological alterations, including almost undetectable B cells with normal total and specific Ig levels, a significant impairment of the helper and cytotoxic naïve T cell compartment associated with impaired T cell proliferation, recurrent respiratory infections with chronic lung changes, and immune dysregulation supporting a diagnosis of T^low^B^−^NK^+^ CID. This suggests that *ZNF699* alterations should be considered as a potential genetic cause of CID with syndromic features. Considering the variability of the clinical phenotypes described in the literature, cohort studies are ongoing to better define the penetrance and the clinical and laboratory immunological features of this syndrome and to define the role of this gene in the development and function of the immune system. Even though the specific role of *ZNF699* in T and B cell development is currently unknown, an involvement in the development of both the lymphoid and the myeloid compartment has been already described in a few ZF proteins. KRAB-domain ZF proteins are a family of transcriptional repressors that exert their activity through recruitment of KAP1/TRIM28 and the establishment of heterochromatin via SETDB1-mediated H3K9 trimethylation. Variants affecting the KRAB domain are expected to impair this protein–protein interaction interface, potentially weakening the assembly of the KRAB–KAP1 repression complex, possibly leading to widespread epigenomic instability with transcriptional consequences.

Several limitations of this report should be acknowledged. The DNA methylation analysis was performed using the established EpiSign clinical interpretation framework, which compares individual samples against validated disease-specific reference cohorts to identify characteristic episignatures. However, formal cell type deconvolution analysis was not performed on this individual sample, and therefore, the potential contribution of altered blood cell composition to the observed methylation profile cannot be fully excluded. In addition, while the analytical framework used is clinically validated and standardized, not all underlying analytical parameters used by EpiSign software are available. Finally, the broader biological interpretation of differentially methylated regions and associated genes remains hypothesis generating, as direct functional validation was beyond the scope of this single case report. Further studies, including larger cohorts, mechanistic models, and high-resolution single-cell approaches, are needed to define DEGCAGS immunopathogenesis.

## Materials and methods

Written informed consent was obtained from the patient for the publication of the case. Genome-wide methylation profiling was performed using DNA extracted from peripheral blood and processed with the EpiSign assay at the Verspeeten Clinical Genome Centre (Canada) as previously described (5). Bisulfite-converted DNA was hybridized to Illumina Infinium EPIC V2 arrays. As part of the standard EpiSign clinical workflow, sample-level quality assessment included evaluation of β-value distribution, overall data structure, probe failure rate, and sample fidelity through age and sex prediction. β values were calculated (0–1 scale) and analyzed using a support vector machine classifier trained on >20,000 reference profiles. To account for potential confounding by leukocyte heterogeneity, blood cell counts were incorporated as covariates during normalization. Disorder-specific MVP scores >0.5 were considered positive. Structural modeling of ZNF699 variants was performed using AlphaFold2 and visualized with UCSF Chimera 1.18. Functional impact prediction was assessed with AlphaMissense.

## Data Availability

The datasets for this article are not publicly available due to concerns regarding participant/patient anonymity. Requests to access the datasets should be directed to the corresponding author.
